# Role of the Anterior Cruciate Ligament, Anterolateral Complex, and Lateral Meniscus Posterior Root in Anterolateral Rotatory Knee Instability: A Biomechanical Study

**DOI:** 10.1177/03635465231161071

**Published:** 2023-03-14

**Authors:** Lukas Willinger, Kiron K. Athwal, Sander Holthof, Andreas B. Imhoff, Andy Williams, Andrew A. Amis

**Affiliations:** * Klinikum rechts der Isar, Technical University of Munich, Munich, Germany; † Imperial College London, London, UK; ‡ Fortius Clinic, London, UK; Investigation performed at Imperial College London, London, UK

**Keywords:** anterior cruciate ligament, anterolateral ligament, Kaplan fibers, lateral meniscus root, kinematics, instability

## Abstract

**Background:**

Injuries to the anterior cruciate ligament (ACL), Kaplan fibers (KFs), anterolateral capsule/ligament (C/ALL), and lateral meniscus posterior root (LMPR) have been separately linked to anterolateral instability.

**Purpose:**

To investigate the contributions of the ACL, KFs, C/ALL, and LMPR to knee stability and to measure instabilities resulting from their injury.

**Study Design:**

Controlled laboratory study.

**Methods:**

Ten fresh-frozen human knees were tested robotically to determine restraints of knee laxity at 0° to 90° of flexion. An 88-N anterior-posterior force (anterior and posterior tibial translation), 5-N·m internal-external rotation, and 8-N·m valgus-varus torque were imposed and intact kinematics recorded. The kinematics were replayed after sequentially cutting the structures (order varied) to calculate their contributions to stability. Another 10 knees were tested in a kinematics rig with optical tracking to measure instabilities after sequentially cutting the structures across 0° to 100° of flexion. One- and 2-way repeated-measures analyses of variance with Bonferroni correction were used to find significance (*P* < .05) for the robotic and kinematics tests.

**Results:**

The ACL was the primary restraint for anterior tibial translation; other structures were insignificant (<10% contribution). The KFs and C/ALL resisted internal rotation, reaching 44% ± 23% (mean ± SD; *P* < .01) and 14% ± 13% (*P* < .05) at 90°. The LMPR resisted valgus but not internal rotation. Anterior tibial translation increased after ACL transection (*P* < .001) and after cutting the lateral structures from 70° to 100° (*P* < .05). Pivot-shift loading increased anterolateral rotational instability after ACL transection from 0° to 40° (*P* < .05) and further after cutting the lateral structures from 0° to 100° (*P* < .01).

**Conclusion:**

The anterolateral complex acts as a functional unit to provide rotatory stability. The ACL is the primary stabilizer for anterior tibial translation. The KFs are the most important internal rotation restraint >30° of flexion. Combined KFs + C/ALL injury substantially increased anterolateral rotational instability while isolated injury of either did not. LMPR deficiency did not cause significant instability with the ACL intact.

**Clinical Relevance:**

This study is a comprehensive biomechanical sectioning investigation of the knee stability contributions of the ACL, anterolateral complex, and LMPR and the instability after their transection. The ACL is significant in controlling internal rotation only in extension. In flexion, the KFs are dominant, synergistic with the C/ALL. LMPR tear has an insignificant effect with the ACL intact.

Anterior cruciate ligament (ACL) tears are frequently accompanied by injuries to the anterolateral complex, including the capsule and anterolateral ligament (C/ALL) in 51% to 76% of ACL tears, Kaplan fibers (KFs) in 19% to 85%, and the lateral meniscus posterior root (LMPR) in 30% to 40%.^11,16,18,31^ As compared with isolated ACL injuries, these concomitant lesions are associated with higher grades of anterolateral knee instability and the pivot-shift phenomenon.^9,25,30,31,43^ Injuries to the KFs, the femoral attachment of the capsulo-osseous layer of the iliotibial band (ITB), the anterolateral ligament (ALL), and the LMPR have each been identified to increase anterolateral knee instability, clinically and in vitro.^13,14,26,29^

Persistent knee anterolateral rotational instability (ALRI) is related to inferior clinical results and return to sports.^4,41^ Therefore, there has been a change in the treatment paradigm of ACL injuries toward not only dealing with the ACL itself but seeking and repairing peripheral capsular and meniscal injuries more carefully. Biomechanical studies have shown that anterolateral procedures can help to restore knee kinematics better than can isolated ACL reconstruction in combined injuries.^14,22,23^ Clinically, this is reflected by a reduced ACL graft failure rate and fewer secondary meniscal lesions.^38,39^ However, one study suggested that these procedures should be performed only if anterolateral structures are substantially injured.^17^ It is vital to know the relative importance of the soft tissue structures for knee stability so that their surgery may be prioritized appropriately. That is why it is crucial to identify the contributions of each of them to knee stability (ie, how much each structure restrains tibiofemoral subluxation) and to quantify their effect on knee instability (ie, how much the tibiofemoral joint laxity increases above intact values) after injury.

Therefore, the aims of this study were to quantify the relative contributions of the ACL, C/ALL, KFs, and LMPR to translational and rotatory stability of the knee and to measure the increase in translational and rotatory knee instabilities after transecting the ACL, C/ALL, KFs, and LMPR.

The following was hypothesized: (1) the ACL is the main restraint to anterior tibial translation (ATT), while the KFs are the main restraints of internal rotation (IR); (2) there is significant ATT instability after cutting the ACL; and (3) the highest rise in IR and simulated pivot-shift (SPS) instability occurs after sectioning the KFs.

## Methods

After ethics approval (Imperial College Healthcare Tissue Bank project R18027-5A), 22 unpaired fresh-frozen human cadaveric knees (13 male, 9 female; mean age, 57 years [range, 47-65 years]) were obtained from a tissue bank (Medcure). They were stored at −20°C and thawed for 24 hours at room temperature before preparation. They had no evidence of previous surgery, abnormal laxity, ligament or meniscal damage, articular erosions, or malalignment via manual examination and arthroscopic visualization by an orthopaedic surgeon (L.W.). Ten knees were tested robotically to obtain the contributions to stability, and 12 knees were tested kinematically to measure the resulting instability. However, 2 knees had to be excluded from the kinematics testing owing to technical error, leaving 10 for analysis. Knees were kept moist with intermittent water spraying during the entire test.

### Specimen Preparation

The femora and tibiae were cut 170 mm from the joint line. Skin and subcutaneous fat were removed for the robotic testing but not for the kinematics testing. All other soft tissues on the femur and tibia within 80 mm from the joint line were left intact. Tissue attaching more proximally and distally was removed, exposing the bones for fixation. The fibula was shortened and secured to the tibia in its anatomic position using a tricortical bone screw.

### Robotic Testing

Ten cadaveric knees were used to determine the contribution of each structure to knee stability. The ends of the femora and the tibiae were embedded into steel pots using bone cement for rigid mounting onto the robotic testing system (Stäubli TX90; Stäubli AG) equipped with a 6-axis universal force-moment sensor (Omega 85; ATI Industrial Automation). The robot had repeatability of 0.03 mm in translation (manufacturer’s specification). The sensor had a force-sensing resolution <0.44 N and torque-sensing resolution <0.014 N·m. The femur was attached to the stationary base of the robot, and the tibia was attached to the load sensor on the moving end effector of the robotic arm.^26^ Before testing, the knees were manually flexed 20 times from 0° to 120° to minimize tissue hysteresis.

The passive path of each knee was determined from 0° to 90° of flexion by minimizing all constraining forces and torques in all other 5 degrees of freedom. Subsequently, ±88-N anterior-posterior force, ±5-N·m IR–external rotation (IR-ER) torque, and ±8-N·m valgus-varus torque were imposed to record the native knee laxity in full extension and 30°, 60°, and 90° of flexion as the datum for the following tests. The movements of the intact knee were replayed after sequentially transecting each structure of interest, and the drop in force/torque to execute the movement after each cut was measured as a reflection of a structure’s ability to resist that motion. This represented the contribution of the transected structure to knee stability, using the principle of superposition.^45^

### Kinematics Rig Testing

Ten more specimens were used to measure knee laxity changes after ligament transection in a 6 degrees of freedom kinematics rig as described previously.^22,44^ The femur was cemented into a cylindrical pot with an anatomic 6° of valgus offset, while the tibia was cemented into a pot with a 500-mm axial extension rod for applying rotational torques. The femoral pot was fixed to the kinematics rig with the transepicondylar axis aligned to the flexion axis of the rig. The rig allowed passive motion of the femur from 0° to 110° of flexion while the tibia hung vertically and unrestricted. This allowed loads simulating clinical evaluations of knee stability to be applied to the tibia across the arc of flexion.

A 5.5-mm Steinmann pin was drilled mediolaterally through the proximal tibia, and 2 semicircular metal hoops were mounted on it. These were used to apply 88-N anterior-posterior translation forces via a string, pulley, and hanging weights with unconstrained IR-ER. Additionally, a 250-mm polyethylene pulley on the tibial extending rod allowed the application of 5-N·m IR-ER as well as 8-N·m varus-valgus torques using a string-and-pulley system. The SPS test used a combined load of 5-N·m IR and 8-N·m valgus torques.

Knee laxity kinematics were measured using an optical tracking system, including a Polaris camera system (NDI) and BrainLab reflective markers (Brainlab, Munich), with a root mean squared translational accuracy of ±0.12 mm (NDI specification). The markers were firmly fixed to the femur and tibia using bicortical rods. The medial and lateral epicondyles, the proximal end of the femur, the most medial and lateral points of the tibial plateau, and the distal end of the tibia were marked with small fiducial marker screws. These were digitized using a stylus probe to define the femoral and tibial coordinate systems, and 0° of flexion was defined as when the tibial and femoral pots were parallel in the sagittal plane. Six degrees of freedom motion was then measured as the tibial movement relative to the femur. The kinematics of the knee were measured across 0° to 100° of flexion-extension for 3 movement cycles while each load was applied. All kinematic data were calculated and presented as changes of the motion from that of the intact knee neutral path of motion, when the joint had no extra loads imposed on it. These changes of motion were initially the native joint laxity and then the instabilities caused by tissue transection, as defined earlier.

### Cutting Sequences

Sequential transections of the ACL, KFs, C/ALL, and the LMPR were performed while the knee remained in the robot or kinematics rig at 90° of flexion:

1. The ACL was transected arthroscopically at the midsubstance using a scalpel introduced through an anteromedial portal and was visually confirmed arthroscopically.
2. The KFs were identified through a lateral approach that split the superficial ITB and then separated proximal and distal bundles from their lateral femoral attachments.
3. The anterolateral capsule including ALL fibers was transected using a cut parallel and anterior to the lateral collateral ligament from its femoral attachment to 10 mm below the joint line anterior to the fibular head—the anterolateral capsular fibers and ALL pass across this cutting line with the knee flexed to 90°.
4. The LMPR tear was simulated via transection of the posterior lateral meniscus lateral to the attachments of the meniscofemoral ligaments to create a worst-case scenario.^11^ For the robotic study, the cut was made using a small vertical posterior approach and verified arthroscopically. In the kinematics rig, the LMPR was transected arthroscopically through an anterolateral portal.

After testing 5 knees in the robot, a significant contribution of the LMPR to resist valgus rotation was found. After that analysis, a superficial medial collateral ligament (sMCL) transection was added to the last 5 knees to compare the sMCL restraint with the LMPR. The cutting sequence was reversed after this point, to overcome any bias owing to the cutting order. Also, 2 additional cuts were recorded, and their effects (not significant, <5%) were subtracted from subsequent data: a split of the superficial ITB along its fibers to access the KFs and capsule and a 5-mm vertical split of the posterolateral capsule to approach the LMPR.

With the kinematics rig, the testing and cutting order was as follows:

- Intact
- ACL transected

There were also 3 cutting orders of secondary restraints in an ACL-deficient knee (n = 3 or 4 per group):

- C/ALL, KFs, LMPR
- LMPR, ALL, KFs
- KFs, LMPR, ALL

### Statistics

A prospective power analysis based on previous work^26^ showed that a ligament-stabilizing contribution of 10% could be identified with a power of 0.95 and an alpha of .05 with 9 specimens, using G*Power Version 3.1.9.7 (Heinrih Heine University, Dusseldorf). A similar prospective power analysis based on the work of Inderhaug et al^22^ showed that a 5° change of IR could be identified with a power of 0.80 and an alpha of .05 with 10 specimens.

Data were analyzed using SPSS Version 24.0 (IBM) and are given as mean and standard deviation. Statistical analysis was performed as follows:

- One-way repeated-measures analysis of variance was used to find significance of the contributions of the anatomic structures across the cutting stages separately for each load at each flexion angle (robot testing).
- Two-way repeated-measures analysis of variance with Bonferroni correction was used to find statistical significance of laxity increases across the cutting stages (intact, ACL cut, anterolateral structures cut) and flexion angles for each loading case (kinematics rig testing). The results of the laxity increases after transecting individual secondary restraints were not tested but are descriptively reported.

Statistical significance was set at *P* < .05.

## Results

### Tibial Anterior-Posterior Translation

The ACL was the only structure providing significant restraint of ATT from 0° to 90° of flexion (*P* < .001). This ranged from 94% of the total restraint of the structures examined at 0° of flexion to 88% at 90° of flexion. None of the other structures resisted ATT significantly; the largest contribution was from the KFs, which provided 7% of the restraint at 60° and 90° of flexion (not significant). The LMPR resisted posterior tibial translation at all 4 flexion angles (*P* < .01), with a maximum of 12% at 90° of flexion.

**Figure 1.**
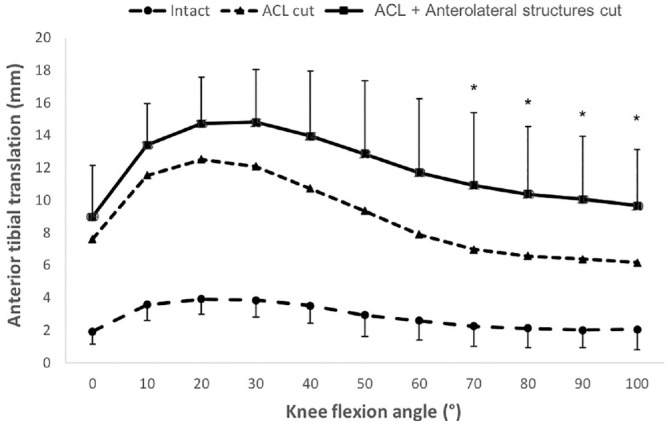
Anterior tibial translation laxity of the intact knee and instability after cutting the ACL and the anterolateral structures in response to 88-N anterior translation force in 6 degrees of freedom kinematics rig testing. The anterior translation was significantly increased after the ACL was cut at all flexion angles (*P* < .001). Cutting the anterolateral structures also significantly increased knee laxity from 70° to 100°. *P < .05. Data are presented as mean ± SD (n = 10). ACL, anterior cruciate ligament.

Transecting the ACL caused significant anterior translation instability throughout the flexion range (*P* < .001) (Figure 1), up to 9 mm at 20° of flexion. Transection of individual lateral structures (KFs, C/ALL, or LMPR) did not increase ATT significantly beyond the ACL-deficient instability, but combined transection of them all increased the ATT between 70° and 100° of flexion up to 4 mm (*P* < .05). Posterior tibial translation was not affected by transecting the ACL or the lateral knee structures.

### Tibial IR-ER

**Figure 2.**
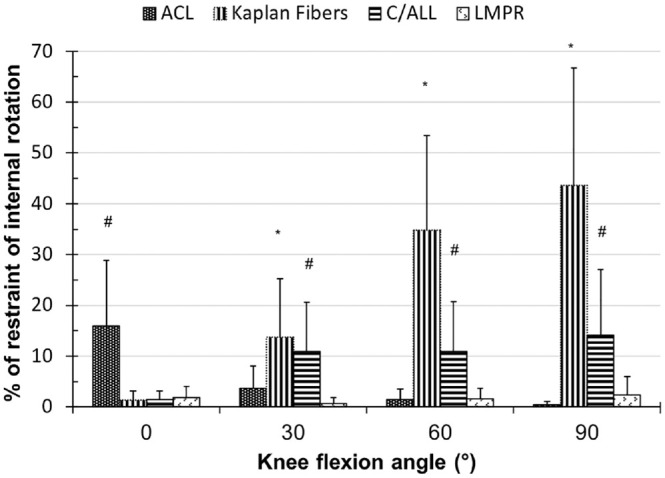
The contribution of the anterior cruciate ligament (ACL), Kaplan fibers, anterolateral capsule including the anterolateral ligament (C/ALL), and the lateral meniscus posterior root (LMPR) to resist internal rotation in robotic testing. ^#^*P* < .05. *P < .01. Data are presented as mean ± SD (n = 10).

IR was resisted mainly by the KFs, reaching 44% ± 23% at 90° of flexion (Figure 2), followed by C/ALL at 14% ± 13% at 90°. The KFs were significantly greater restraints than the C/ALL at 60° and 90° of knee flexion (*P* < .05). The ACL resisted IR in 0° of knee flexion but was insignificant in higher flexion. The LMPR was not a significant restraint of IR in an ACL-intact knee. ER was not restrained significantly by any of the examined structures.

**Figure 3.**
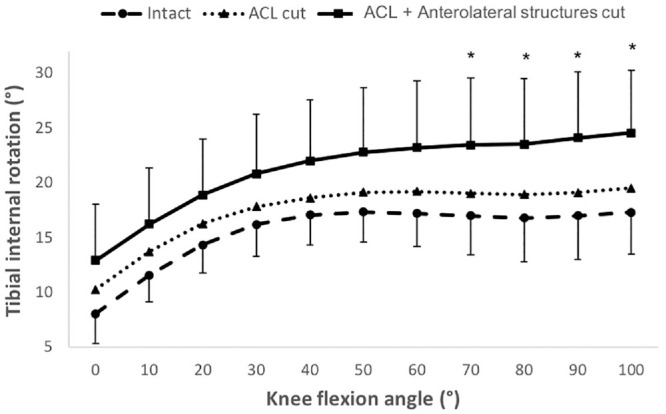
Changes in internal rotation after transecting the ACL and then the anterolateral structures in response to 5-N·m internal rotation torque in 6 degrees of freedom kinematics rig testing. Data are presented as mean ± SD (n = 10). *P < .05 (significant increase above ACL cut state). ACL, anterior cruciate ligament.

IR was not significantly increased at any angle of flexion by isolated transection of the ACL (Figure 3). Additional cutting of the anterolateral structures caused significant IR instability as compared with the isolated ACL cut state from 70° of knee flexion onward. The combined deficient knee (ACL + lateral structures) had significantly greater IR instability than the intact knee had across 0° to 100° of flexion (0° and 40°-100°, *P* < .01; 10°-30°, *P* < .05). Cutting the ACL and lateral structures did not cause significant ER instability.

### Tibial Valgus and Varus Rotation

**Figure 4.**
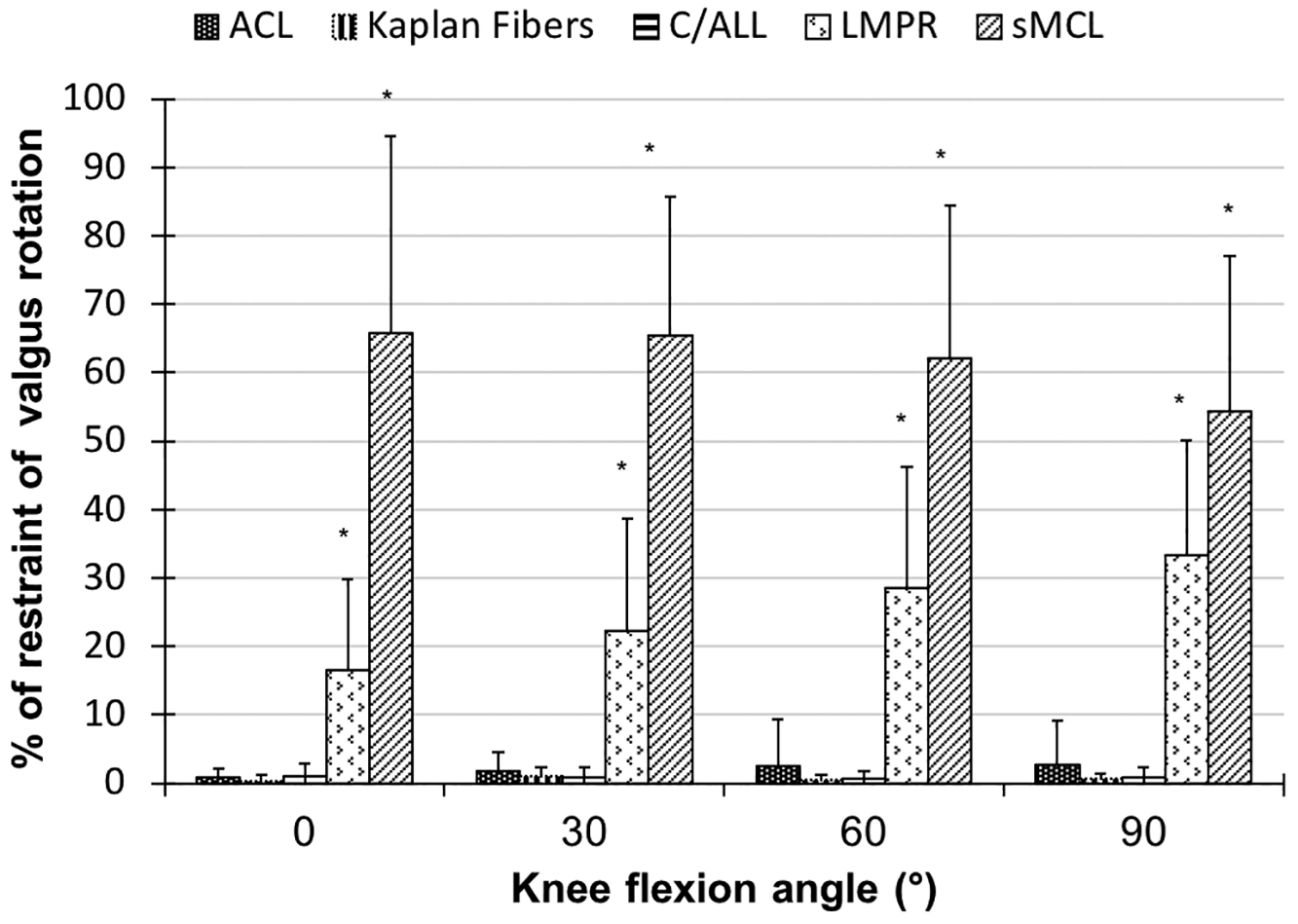
The contribution of the anterior cruciate ligament (ACL), Kaplan fibers, anterolateral capsule including the anterolateral ligament (C/ALL), and the lateral meniscus posterior root (LMPR) to resist valgus rotation in robotic testing. *P < .001. Data are presented as mean ± SD (n = 10, apart from the superficial medial collateral ligament [sMCL] when n = 5).

Transecting the LMPR showed that the lateral meniscus is a significant restraint of valgus rotation (Figure 4). In the 5 knees where the sMCL was transected, it was the highest restraint against valgus rotation (*P* < .001).

None of the investigated structures resisted varus rotation significantly. Cutting the ACL, KFs, ALL, and LMPR did not cause significant varus or valgus instability.

### Simulated Pivot-Shift Instability: Combined IR and Valgus Rotation

#### Anterior Tibial Translation

**Figure 5.**
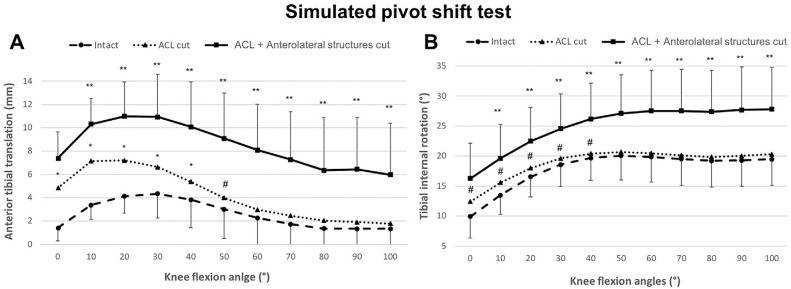
Changes in (A) anterior tibial translation and (B) internal rotation after cutting the anterior cruciate ligament (ACL) and the anterolateral structures in response to simulated pivot-shift load (combined 5-N·m internal torque and 8-N·m valgus torque) in 6 degrees of freedom kinematics rig testing. *P < .01. ^#^*P* < .05 vs intact state. **P < .001 vs ACL cut state. Data are presented as mean ± SD (n = 10).

ACL transection resulted in significant anterior translation instability during the SPS loading across 0° to 50° of flexion (Figure 5A). Transecting the lateral structures led to an additional significant increase in ATT instability from 10° to 100° of flexion (*P* < .001).

#### Internal Rotation

Transecting the ACL caused small IR instability (mean ≤2°) during SPS loading from 0° to 40° of flexion (Figure 5B). Transecting the lateral structures caused a larger increase in IR instability, significant from 10° to 100° of flexion and averaging 7° across 50° to 100° of flexion; up to 5° of this increase followed transection of the KFs. The combined injured knee (ACL + lateral structures) had significant IR instability at all flexion angles, reaching an 8° increase above native laxity at 100° of flexion.

**Figure 6.**
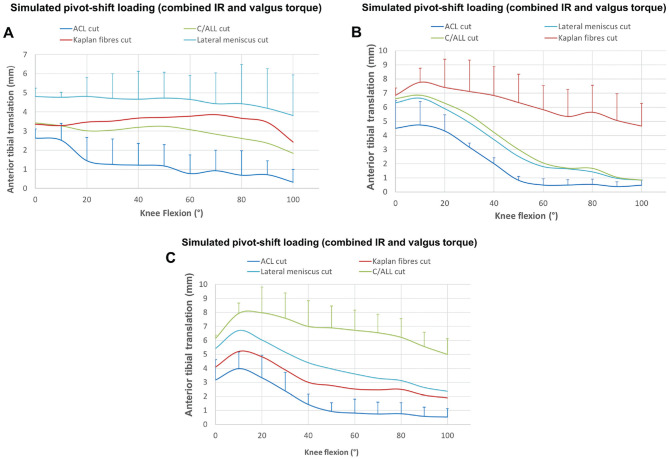
The resulting anterior tibial translation in response to a simulated pivot shift (combined 5-N·m internal torque and 8-N·m valgus torque) in 3 cutting orders: (A) ACL, C/ALL, KFs, LMPR (n = 3); (B) ACL, LMPR, C/ALL, KFs (n = 3); and (C) ACL, KFs, LMPR, C/ALL (n = 4). ACL, anterior cruciate ligament; C/ALL, anterolateral capsule and ligament; KFs, Kaplan fibers; LMPR, lateral meniscus posterior root.

**Figure 7.**
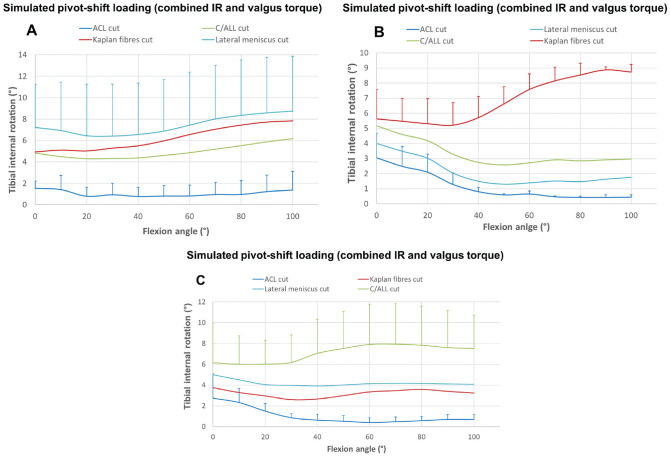
The resulting tibial internal rotation in response to a simulated pivot shift (combined 5-N·m internal torque and 8-N·m valgus torque) in 3 cutting orders: (A) ACL, C/ALL, KFs, LMPR (n = 3); (B) ACL, LMPR, C/ALL, KFs (n = 3); and (C) ACL, KFs, LMPR, C/ALL (n = 4). ACL, anterior cruciate ligament; C/ALL anterolateral capsule and ligament; KFs, Kaplan fibers; LMPR, lateral meniscus posterior root.

Figure 6 (for ATT) and Figure 7 (for IR) show the increased instability during SPS loading resulting from transecting the individual structures in 3 orders. Although it is difficult to make firm conclusions when the sample size is 3 or 4 per group, these graphs show that the largest increases of instability were associated with specific structures being the last one to be transected for the KFs (graphs B in Figures 6 and 7) and C/ALL (graphs C in Figures 6 and 7) for ATT and IR. The exception to this was the increased ATT near knee extension when the ACL was transected, when it was the primary restraint.

## Discussion

This study showed that substantial ALRI instability with SPS loading is seen only when the KFs and C/ALL are transected and not with isolated deficiency of either one, indicating that they act synergistically to restrain ALRI. The KFs are the most important restraint of IR in higher flexion angles. Similarly, the ACL is the primary restraint of ATT and resists IR in the extended knee. These findings are more subtle than the original hypotheses and reconcile previously conflicting reports. They have arisen from using robotic and kinematic methods in a single study to obtain complementary restraint and instability data. When these data are taken as a whole, they show how the ACL and the lateral structures work together across the range of flexion.

The controversy regarding the restraints to ALRI still causes lively discussion within the orthopaedic community. The functions of the anterolateral knee structures have been widely investigated, but previous results differed. Injuries to the KFs, C/ALL, and LMPR have each been linked to rotatory instability, with different authors advocating one or another structure to be more important.^13,21,26,27^ Furthermore, some studies focused on structure while not investigating the others, and some knees were ACL intact while others were ACL deficient, which could overestimate the importance of those structures that were studied.^12,30,35,36^ At least part of this controversy has arisen from measuring only changes of knee instability (laxity), which is observed clinically but is not the same as assessing the importance of structures as restraints of joint laxity, which provide stability of the knee. The additional restraint data result from robotic testing in which the forces and torques acting on the knee are measured when it is tested. The graphs of ATT and IR (Figures 6 and 7) demonstrate that instabilities related to each anatomic structure are cutting sequence dependent and so cannot be used to discern the restraint provided by each structure.

The ACL has been confirmed to be the primary restraint of ATT, similar to previous studies, and this is well known.^6,26^ The restraint to ATT provided by the lateral structures studied was insignificant: the largest contribution, from the KFs in the flexed knee, was only 7% of the total. Transecting the ACL led to significant anterior translation instability in all flexion angles and in SPS testing at lower flexion angles, which reflects the sensitivity of the Lachman test as compared with the anterior drawer test. Transecting the other structures had the largest effect on anterior translation instability in the flexed knee, typically increasing ATT from 6 to 10 mm.

The role of the ACL in controlling rotational instability is less well understood: it was described as a primary stabilizer to IR in earlier studies.^10,27^ However, a growing body of evidence shows that the ACL has only a minor role in controlling IR, principally near full extension.^2,3,26,28,32^ The present study shows that the ACL contributes a maximum of 16% of the resistance to IR. By 30° of flexion, the anterolateral soft tissues are more important in resisting IR, and the ACL is insignificant. This results from their longer lever arm about the axis of tibial IR-ER when compared with the central ACL and their more efficient orientation to resist IR with knee flexion.

The restraint of IR provided by the KF attachments of the ITB on the distal lateral femur was described in 1958.^24^ Around 50% of ITB injuries occurring with ACL rupture are at the femoral KF attachments,^5,7,8^ so this clinical injury pattern was simulated, in contrast to previous studies that transected the ITB.^21,26,37^ In the present study, the KFs with the deep ITB were the main restraint of IR across 30° to 90° of flexion, up to 44% at 90° of flexion. Kittl et al^26^ transected the superficial and deep ITB separately and found a higher contribution of the whole ITB in resisting IR at 60° and 90° of flexion of 76% and 72%, respectively. These numbers imply significant IR instability if the ITB is injured in isolation, but only 1° to 3° increases in the flexed knee were reported.^13,21^ These changes of IR would be difficult to find during clinical examination, suggesting that the KFs may not be damaged in isolation. Terry et al^42^ correlated deep ITB injury in ACL-deficient knees with higher pivot-shift instability. In contrast, recent clinical studies did not find a direct association between KF injury on magnetic resonance imaging and a higher grade of pivot-shift test.^5,7,8^ This study shows that ALRI increases substantially after transecting the KFs, up to 5° of IR in SPS. The effect increases with knee flexion and is smaller if the C/ALL are intact.

Many studies have investigated the C/ALL as a stabilizer of ALRI.^13,15,17,21,26,33,37^ One in vitro study showed that the C/ALL transmitted forces like a sheet of tissue rather than acting as a distinct ligament and should therefore be considered as a whole.^15^ For this reason, we transected the C/ALL as one. Kittl et al^26^ tested these structures separately, finding that the capsule and ALL did not resist IR and SPS significantly. In agreement with this previous report, the present study shows that the C/ALL restrains IR less than the KFs/ITB. Both studies found that the C/ALL complex resists 10% to 15% of IR torque from 30° to 90° of flexion, but there are conflicting opinions whether the C/ALL has a significant role. In an ACL-intact knee, C/ALL deficiency does not cause a significant increase in IR^21,27,37^; thus, it is not a primary stabilizer. In ACL-deficient knees, a small but significant effect on IR after transecting the C/ALL has been reported.^13,34,37,40^ This study supports these previous findings: transecting the C/ALL leads to a small increase of ALRI and IR with the KFs intact and a larger increase of IR instability when the KFs were already transected. The SPS tests found small IR instability when the C/ALL or KFs were transected and then greater IR instability when the other structure was transected. Therefore, considerable IR instability suggests the deficiency of both structures, and if one is intact, then IR is close to the intact state. Note, however, that these IR laxity results are from simulating manual clinical tests, rather than functional loading. The robotic testing in this study and that of Kittl et al^26^ measured the restraint provided by the structures, showing that while the C/ALL plays a role in controlling ALRI, it is less important than the KFs/deep ITB.

This study shows that the lateral meniscus does not resist IR in an ACL-intact knee, but LMPR injury in an ACL-deficient knee leads to a further increase in ALRI. The role of the lateral meniscus in controlling rotatory knee laxity is not well understood. The loose capsular attachment allows mobility on the lateral tibial plateau. This explains why the lateral meniscus did not resist IR in an ACL-intact knee in our robotic test setup. However, there was a high contribution of the lateral meniscus in resisting valgus rotation, increasing with knee flexion as the meniscus supported the femoral condyle. Previous cadaveric studies have reported that a lateral meniscectomy or transection of the LMPR increases IR or ALRI instability.^12,29-31,35,36^ Yet, those reports relate to ACL-deficient knees. We are not aware of work showing that the LMPR controls IR when the ACL is intact. It follows that IR instability persisting after ACL reconstruction means that structures other than, or in addition to, the LMPR are injured and that isolated LMPR repair will not correct it. The findings of the previous studies are in line with the present work, with a small increase of IR when the LMPR is transected in an ACL-deficient knee. Ahn et al^1^ reported that lateral meniscectomy led to increased valgus instability, which supports the finding of the present study. In clinical studies, lateral meniscal injuries have been related to a higher grade of instability in pivot-shift loading.^19,20,31^ These results, combined with contact pressure considerations, suggest that lateral meniscal injuries such as LMPR tears should be addressed during surgery.

The findings of this study arise from cadaveric work and thus have limitations to their clinical translation, but the methods used are based on extensive literature that supports the validity of using (1) fresh-frozen collagenous tissues, (2) sequential cutting studies of restraint in robotic tests, and (3) kinematics to measure increased joint instability. The specimens were older than are typical for ACL injuries, and that may have affected their behavior. This reflects the unavailability of younger specimens, but careful examination ensured a lack of pathologic changes. The structures studied are all passive restraints, and the knees were loaded only to simulate clinical manual examination, assuming that the muscles were relaxed. This does not mean that the joint was distracted during measurements of instability, because the soft tissues crossing the joint were tensed as they resisted the displacing loads, which had much larger effects than the weight of the tibia. While that relates directly to clinical diagnosis of injuries, the relative importance of each structure may differ when the knee is acted on by the muscles and at functional loading. This may affect the interpretation of how these results may manifest in a clinical scenario. Also, the “simulated pivot shift” was a quasi-static test rather than the dynamic clinical maneuver. While this is an established model, a dynamic testing setup might better replicate an actual pivot shift. Against these limitations may be set the ability to perform sequential transections of the structures in vitro and thus to have accurate knowledge of exactly what pathology is present, enabling powerful repeated-measures statistical analysis. Finally, while these findings offer a rationale for surgical interventions to address injured lateral structures, this work did not assess whether surgery can restore stability to the level of the intact knee.

## Conclusion

The anterolateral complex acts as a functional unit to provide rotatory stability. The ACL is the primary stabilizer for ATT. The KFs are the most important IR restraint >30° of flexion. Combined KFs + C/ALL injury substantially increases ALRI, while isolated injury of either does not. LMPR deficiency does not cause significant instability with the ACL intact.

## References

[1] Ahn JH Koh IJ McGarry MH , et al. Knee laxity in anterolateral complex injuries versus lateral meniscus posterior horn injuries in anterior cruciate ligament deficient knees: a cadaveric study. Knee. 2020;27(2):363-374. 31874819 10.1016/j.knee.2019.11.018

[2] Amis AA Scammell BE . Biomechanics of intra-articular and extra-articular reconstruction of the anterior cruciate ligament. J Bone Joint Surg Br. 1993;75(5):812-817. 8376447 10.1302/0301-620X.75B5.8376447

[3] Andersen HN Dyhre-Poulsen P . The anterior cruciate ligament does play a role in controlling axial rotation in the knee. Knee Surg Sports Traumatol Arthrosc. 1997;5(3):145-149. 9335025 10.1007/s001670050042

[4] Ayeni OR Chahal M Tran MN Sprague S . Pivot shift as an outcome measure for ACL reconstruction: a systematic review. Knee Surg Sports Traumatol Arthrosc. 2012;20(4):767-777. 22218828 10.1007/s00167-011-1860-y

[5] Balendra G Willinger L Pai V , et al. Anterolateral complex injuries occur in the majority of “isolated” anterior cruciate ligament ruptures. Knee Surg Sports Traumatol Arthrosc. 2022;30(1):176-183. 33796903 10.1007/s00167-021-06543-6

[6] Ball S Stephen JM El-Daou H Williams A Amis AA . The medial ligaments and the ACL restrain anteromedial laxity of the knee. Knee Surg Sports Traumatol Arthrosc. 2020;28(12):3700-3708. 32504158 10.1007/s00167-020-06084-4 PMC7669770

[7] Batty LM Murgier J Feller JA O’Sullivan R Webster KE Devitt BM . Radiological identification of injury to the Kaplan fibers of the iliotibial band in association with anterior cruciate ligament injury. Am J Sports Med. 2020;48(9):2213-2220. 32579396 10.1177/0363546520931854

[8] Berthold DP Willinger L LeVasseur MR , et al. High rate of initially overlooked Kaplan fiber complex injuries in patients with isolated anterior cruciate ligament injury. Am J Sports Med. 2021;49(8): 2117-2124. 34086492 10.1177/03635465211015682 PMC8246408

[9] Cavaignac E Faruch M Wytrykowski K , et al. Ultrasonographic evaluation of anterolateral ligament injuries: correlation with magnetic resonance imaging and pivot-shift testing. Arthroscopy. 2017;33(7): 1384-1390. 28343806 10.1016/j.arthro.2017.01.040

[10] Engebretsen L Wijdicks CA Anderson CJ Westerhaus B LaPrade RF . Evaluation of a simulated pivot shift test: a biomechanical study. Knee Surg Sports Traumatol Arthrosc. 2012;20(4):698-702. 22057355 10.1007/s00167-011-1744-1

[11] Forkel P Reuter S Sprenker F , et al. Different patterns of lateral meniscus root tears in ACL injuries: application of a differentiated classification system. Knee Surg Sports Traumatol Arthrosc. 2015; 23(1):112-118. 25502611 10.1007/s00167-014-3467-6

[12] Forkel P von Deimling C Lacheta L , et al. Repair of the lateral posterior meniscal root improves stability in an ACL-deficient knee. Knee Surg Sports Traumatol Arthrosc. 2018;26(8):2302-2309. 29704113 10.1007/s00167-018-4949-8

[13] Geeslin AG Chahla J Moatshe G , et al. Anterolateral knee extra-articular stabilizers: a robotic sectioning study of the anterolateral ligament and distal iliotibial band Kaplan fibers. Am J Sports Med. 2018;46(6):1352-1361. 29558208 10.1177/0363546518759053

[14] Guenther D Irarrazaval S Bell KM , et al. The role of extra-articular tenodesis in combined ACL and anterolateral capsular injury. J Bone Joint Surg Am. 2017;99(19):1654-1660. 28976430 10.2106/JBJS.16.01462

[15] Guenther D Rahnemai-Azar AA Bell KM , et al. The anterolateral capsule of the knee behaves like a sheet of fibrous tissue. Am J Sports Med. 2017;45(4):849-855. 27932332 10.1177/0363546516674477

[16] Helito CP Helito PVP Costa HP Demange MK Bordalo-Rodrigues M . Assessment of the anterolateral ligament of the knee by magnetic resonance imaging in acute injuries of the anterior cruciate ligament. Arthroscopy. 2017;33(1):140-146. 27324971 10.1016/j.arthro.2016.05.009

[17] Herbst E Arilla FV Guenther D , et al. Lateral extra-articular tenodesis has no effect in knees with isolated anterior cruciate ligament injury. Arthroscopy. 2018;34(1):251-260. 29079261 10.1016/j.arthro.2017.08.258

[18] Herbst E Hoser C Tecklenburg K , et al. The lateral femoral notch sign following ACL injury: frequency, morphology and relation to meniscal injury and sports activity. Knee Surg Sports Traumatol Arthrosc. 2015;23(8):2250-2258. 24797811 10.1007/s00167-014-3022-5

[19] Hoshino Y Miyaji N Nishida K , et al. The concomitant lateral meniscus injury increased the pivot shift in the anterior cruciate ligament–injured knee. Knee Surg Sports Traumatol Arthrosc. 2019;27(2):646-651. 30310925 10.1007/s00167-018-5209-7

[20] Hosseini A Li JS Gill TJT Li G . Meniscus injuries alter the kinematics of knees with anterior cruciate ligament deficiency. Orthop J Sports Med. 2014;2(8):2325967114547346. 10.1177/2325967114547346 PMC4555577 26535357

[21] Huser LE Noyes FR Jurgensmeier D Levy MS . Anterolateral ligament and iliotibial band control of rotational stability in the anterior cruciate ligament–intact knee: defined by tibiofemoral compartment translations and rotations. Arthroscopy. 2017;33(3):595-604. 27964969 10.1016/j.arthro.2016.08.034

[22] Inderhaug E Stephen JM Williams A Amis AA . Anterolateral tenodesis or anterolateral ligament complex reconstruction: effect of flexion angle at graft fixation when combined with ACL reconstruction. Am J Sports Med. 2017;45(13):3089-3097. 28898106 10.1177/0363546517724422

[23] Inderhaug E Stephen JM Williams A Amis AA . Biomechanical comparison of anterolateral procedures combined with anterior cruciate ligament reconstruction. Am J Sports Med. 2017;45(2):347-354. 28027653 10.1177/0363546516681555

[24] Kaplan EB . The iliotibial tract: clinical and morphological significance. J Bone Joint Surg Am. 1958;40(4):817-832. 13549519

[25] Khanna M Gupte C Dodds A Williams A Walker M . Magnetic resonance imaging appearances of the capsulo-osseous layer of the iliotibial band and femoral attachments of the iliotibial band in the normal and pivot-shift ACL injured knee. Skeletal Radiol. 2019; 48(5):729-740. 30593591 10.1007/s00256-018-3128-9 PMC6456473

[26] Kittl C El-Daou H Athwal KK , et al. The role of the anterolateral structures and the ACL in controlling laxity of the intact and ACL-deficient knee. Am J Sports Med. 2016;44(2):345-354. 26657572 10.1177/0363546515614312

[27] Lipke JM Janecki CJ Nelson CL , et al. The role of incompetence of the anterior cruciate and lateral ligaments in anterolateral and anteromedial instability: a biomechanical study of cadaver knees. J Bone Joint Surg Am. 1981;63(6):954-960. 7240336

[28] Lorbach O Pape D Maas S , et al. Influence of the anteromedial and posterolateral bundles of the anterior cruciate ligament on external and internal tibiofemoral rotation. Am J Sports Med. 2010;38(4): 721-727. 20200323 10.1177/0363546509353133

[29] Lording T Corbo G Bryant D Burkhart TA Getgood A . Rotational laxity control by the anterolateral ligament and the lateral meniscus is dependent on knee flexion angle: a cadaveric biomechanical study. Clin Orthop Relat Res. 2017;475(10):2401-2408. 28536855 10.1007/s11999-017-5364-z PMC5599389

[30] Musahl V Citak M O’Loughlin PF Choi D Bedi A Pearle AD . The effect of medial versus lateral meniscectomy on the stability of the anterior cruciate ligament–deficient knee. Am J Sports Med. 2010;38(8):1591-1597. 20530720 10.1177/0363546510364402

[31] Musahl V Rahnemai-Azar AA Costello J , et al. The influence of meniscal and anterolateral capsular injury on knee laxity in patients with anterior cruciate ligament injuries. Am J Sports Med. 2016;44(12):3126-3131. 27507843 10.1177/0363546516659649

[32] Oh YK Kreinbrink JL Ashton-Miller JA Wojtys EM . Effect of ACL transection on internal tibial rotation in an in vitro simulated pivot landing. J Bone Joint Surg Am. 2011;93(4):372-380. 21325589 10.2106/JBJS.J.00262 PMC3033203

[33] Parsons EM Gee AO Spiekerman C Cavanagh PR . The biomechanical function of the anterolateral ligament of the knee: response. Am J Sports Med. 2015;43(8):NP22. 10.1177/0363546515597218 26232458

[34] Rasmussen MT Nitri M Williams BT , et al. An in vitro robotic assessment of the anterolateral ligament, part 1: secondary role of the anterolateral ligament in the setting of an anterior cruciate ligament injury. Am J Sports Med. 2016;44(3):585-592. 26684663 10.1177/0363546515618387

[35] Shybut TB Vega CE Haddad J , et al. Effect of lateral meniscal root tear on the stability of the anterior cruciate ligament–deficient knee. Am J Sports Med. 2015;43(4):905-911. 25589386 10.1177/0363546514563910

[36] Smith PA Bezold WA Cook CR , et al. Kinematic analysis of lateral meniscal oblique radial tears in the anterior cruciate ligament–deficient knee. Am J Sports Med. 2021;49(14):3898-3905. 34699272 10.1177/03635465211052521

[37] Sonnery-Cottet B Lutz C Daggett M , et al. The involvement of the anterolateral ligament in rotational control of the knee. Am J Sports Med. 2016;44(5):1209-1214. 26865395 10.1177/0363546515625282

[38] Sonnery-Cottet B Saithna A Blakeney WG , et al. Anterolateral ligament reconstruction protects the repaired medial meniscus: a comparative study of 383 anterior cruciate ligament reconstructions from the SANTI Study Group with a minimum follow-up of 2 years. Am J Sports Med. 2018;46(8):1819-1826. 29741400 10.1177/0363546518767659

[39] Sonnery-Cottet B Saithna A Cavalier M , et al. Anterolateral ligament reconstruction is associated with significantly reduced ACL graft rupture rates at a minimum follow-up of 2 years: a prospective comparative study of 502 patients from the SANTI Study Group. Am J Sports Med. 2017;45(7):1547-1557. 28151693 10.1177/0363546516686057

[40] Spencer L Burkhart TA Tran MN , et al. Biomechanical analysis of simulated clinical testing and reconstruction of the anterolateral ligament of the knee. Am J Sports Med. 2015;43(9):2189-2197. 26093007 10.1177/0363546515589166

[41] Tashman S Collon D Anderson K Kolowich P Anderst W . Abnormal rotational knee motion during running after anterior cruciate ligament reconstruction. Am J Sports Med. 2004;32(4):975-983. 15150046 10.1177/0363546503261709

[42] Terry GC Norwood LA Hughston JC Caldwell KM . How iliotibial tract injuries of the knee combine with acute anterior cruciate ligament tears to influence abnormal anterior tibial displacement. Am J Sports Med. 1993;21(1):55-60. 8427369 10.1177/036354659302100110

[43] Ueki H Katagiri H Otabe K , et al. Contribution of additional anterolateral structure augmentation to controlling pivot shift in anterior cruciate ligament reconstruction. Am J Sports Med. 2019;47(9):2093-2101. 31211590 10.1177/0363546519854101

[44] Willinger L Athwal KK Williams A Amis AA . An anterior cruciate ligament in vitro rupture model based on clinical imaging. Am J Sports Med. 2021;49(9):2387-2395. 34115540 10.1177/03635465211017145 PMC8283191

[45] Woo S L-Y Fisher MB . Evaluation of knee stability with use of a robotic system. J Bone Joint Surg Am. 2009;91(suppl 1):78-84. 19182030 10.2106/JBJS.H.01371 PMC2663353

